# Distribution of Chromophytic Phytoplankton in the Eddy-Induced Upwelling Region of the West Pacific Ocean Revealed Using *rbc*L Genes

**DOI:** 10.3389/fmicb.2021.596015

**Published:** 2021-03-02

**Authors:** Laxman Pujari, Dhiraj Narale, Jinjun Kan, Chao Wu, Guicheng Zhang, Changling Ding, Liuyang Li, Jun Sun

**Affiliations:** ^1^Research Center for Indian Ocean Ecosystem, Tianjin University of Science and Technology, Tianjin, China; ^2^Stroud Water Research Center, Avondale, PA, United States; ^3^School of Life Sciences and Biotechnology, Shanghai Jiao Tong University, Shanghai, China; ^4^College of Marine Science and Technology, China University of Geosciences, Wuhan, China

**Keywords:** West Pacific Ocean, Western boundary currents, upwelling, *rbc*L gene, chromophytic phytoplankton, high throughput sequencing

## Abstract

Marine chromophytic phytoplankton are a diverse group of algae and contribute significantly to the total oceanic primary production. However, the spatial distribution of chromophytic phytoplankton is understudied in the West Pacific Ocean (WPO). In this study, we have investigated the community structure and spatial distribution of chromophytic phytoplankton using RuBisCO genes (Form ID *rbc*L). Our results showed that Haptophyceae, Pelagophyceae, Cyanophyceae, Xanthophyceae, and Bacillariophyceae were the dominant groups. Further, chromophytic phytoplankton can be distinguished between upwelling and non-upwelling zones of the WPO. Surface and 75 m depths of a non-upwelling area were dominated by *Prochlorococcus* strains, whereas chromophytic phytoplankton were homogenously distributed at the surface layer in the upwelling zone. Meanwhile, *Pelagomonas*-like sequences were dominant at DCM (75 m) and 150 m depths of the upwelling zone. Non-metric multidimensional scaling (NMDS) analysis did not differentiate between chromophytic phytoplankton in the upwelling and non-upwelling areas, however, it showed clear trends of them at different depths. Further, redundancy analysis (RDA) showed the influence of physicochemical parameters on the distribution of chromophytic phytoplankton. Along with phosphate (*p* < 0.01), temperature and other dissolved nutrients were important in driving community structure. The upwelling zone was impacted by a decrease in temperature, salinity, and re-supplement of nutrients, where *Pelagomonas*-like sequences outnumbered other chromophytic groups presented.

## Introduction

Marine phytoplankton are accountable for half of the world’s primary production. They are the key players of the global carbon cycle (Falkowski, 1994; Smetacek, 1999). In recent years, research on phytoplankton diversity has evolved at a rapid pace in which molecular tools have increasingly been used in place of more conventional approaches. Consequently, the high-resolution phytoplankton molecular diversity being recovered across different ecosystems has been achieved through characterizing functional genes involved in metabolisms (such as carbon and nitrogen; Samanta and Bhadury, 2014; Wu et al., 2019). Ribulose-1, 5-bisphosphate carboxylase/oxygenase (RuBisCO) enzyme-encoding *rbc*L gene, is an important gene marker for assessing phylogenetic relationships among photosynthetic organisms, as it is mostly found in chloroplasts where photosynthesis occurs (Gielly and Taberlet, 1994). RuBisCO is a rate-limiting enzyme for photosynthetic CO_2_ fixation in phytoplankton. Until now, four different forms of RuBisCO (I, II, III, and IV) have been discovered. Form I is the most abundant (Tabita, 1999), and it is subdivided into four subclasses, i.e., IA, IB, IC, and ID. Generally, these genes are also divided into the terms ‘green’ and ‘red’ lineages. From ID, RuBisCO contains non-green (red lineage) phytoplankton groups such as Bacillariophyceae, Haptophyceae, Pelagophyceae, Cryptophyceae, Chrysophyceae, and Eustigmatophyceae, and these are termed chromophytic phytoplankton (Tabita, 1999). Earlier studies on functional gene *rbc*L form ID have emphasized its importance through gene abundance, expression and have also provided high-resolution community composition of chromophytic phytoplankton in different marine ecosystems (Pichard et al., 1993; Samanta and Bhadury, 2014). As the *rbc*L gene is chloroplast encoded, the chloroplast gene copy number swithin chromophytic phytoplankton differ, particularly in diatoms. Diatoms may comprise of up to 2000 chloroplast-encoding *rbc*L genes in addition to two copies of *rbc*L gene in their genome (Douglas, 1988).

**Figure 1:**
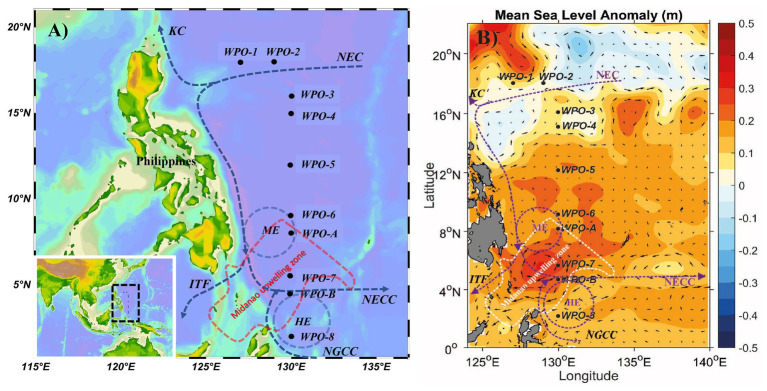
A map showing **(A)** geographic location, and **(B)** mean sea level anomaly (in m) at the sampling sites in the WPO. The mean sea level anomaly is color-coded with scale bar shown on the right side. The major Western Boundary Currents (WBCs) are coded as, the Kuroshio Current (KC), the North Equatorial Current (NEC), the North Equatorial Countercurrent (NECC), the New Guinea Coastal Current (NGCC), the Indonesian Throughflow (ITF) and the Mindanao Eddy (ME), the Halmahera Eddy (HE), and Mindanao upwelling zone are shown on the map (Udarbe-Walker and Villanoy, 2001; Hu et al., 2015; Chen et al., 2018).

The understanding of fundamental factors regulating the distribution of microorganisms including phytoplankton in diverse marine ecosystems is important for the interpretation of global biogeochemistry and climate systems as well as ecological and evolutionary changes (Hanson et al., 2012). The West Pacific Ocean (WPO) is one of the most dynamic and intensive mass water transportation systems in the world (Loder et al., 1998). The WPO possesses various Western Boundary Current (WBC) systems (Figure 1), and these are swift and narrow oceanic currents found in all major oceanic gyre. The North Equatorial Current (NEC) impinges upon the Philippine coast and gives rise to the northward Kuroshio Current (KC) and southward Mindanao Current (MC; Zhang et al., 2017). The KC plays a vital role in the northern Pacific circulation system, which carries warm, saline, and oligotrophic waters. The MC flows southwards along the Philippine coast and further divides to form the North Equatorial Countercurrent (NECC) and Indonesia Throughflow (ITF). The groups of currents known as the Low-Latitude western boundary currents (LLWBCs) and New Guinea Coastal Current/undercurrent (NGCC/NGCCU) flow along New Guinea and meet near Halmahera Island (Kashino et al., 2013). This complex feature of retroflection gives rise to two semipersistent eddies, i.e., the Mindanao Eddy (ME) and Halmahera Eddy (HE). The formation of the Mindanao upwelling system at the retroflection area is a critical physical phenomenon. Such eddy-induced upwelling systems are considered to be the most productive zones of the world ocean (Cushing, 1971). Generally, upwelling zones are recognized by their horizontal anomaly in temperature, salinity, density, oxygen, nutrients, and chlorophyll. The Mindanao eddy upwelling zone is recognized by its cold anomaly at 100 m depth east of Mindanao (Udarbe-Walker and Villanoy, 2001). Although it reaches its maximum size in winter and decreases towards summer, it presents throughout the year with varying sizes (Udarbe-Walker and Villanoy, 2001). Earlier studies elucidated the diversity of chromophytic phytoplankton and their significant contribution in other upwelling zones, including the California coast upwelling zone (Bhadury and Ward, 2009) and Monterey Bay upwelling zone (Paerl et al., 2012). Different currents and upwelling zones in the WPO comprehensively provide a unique environment for the proliferation of the primary producers, including the chromophytic phytoplankton. However, to date, there has been no study carried out to assess the molecular characterization and phylogenetic diversity of chromophytic phytoplankton community structure in the upwelling zone of WPO.

The purpose of the present study was to investigate the community composition of chromophytic phytoplankton in the WPO. Furthermore, this study also assessed the influence of environmental variability on chromophytic phytoplankton community structure in the eddy-induced upwelling region of the WPO using high throughput sequencing of form ID *rbc*L genes.

## Materials and Methods

### Study Area, Sample Collection, and Physicochemical Analysis

The cruise was carried out in the WPO onboard research vessel *KeXue* from October 25th to November 12th 2017 (Figure 1). Samples were collected from 10 different stations, including several vertical depths. Among them, six stations (WPO 1, WPO 2, WPO 3, WPO 4, WPO 5, and WPO 6) were sampled for three depths (0, 75, and 150 m). Due to technical complications, we could not obtain sequences from station “WPO 7” 150 m depth and Station “WPO 8” 0 m and 150 m depths. The other two stations, “WPO A” and “WPO B”, were in the upwelling zone. Therefore, to obtain a comprehensive community structure of chromophytic phytoplankton at the upwelling zone, we collected samples from five different depths (0, 50, 75, 100, and 150 m). Details of the geographical location of stations are drawn in Figure 1A [Ocean Data View (v4.7.7),^1^ 2001; Schlitzer, 2007]. Figure 1B indicates the mean sea level anomaly (m) variations at sampling stations. Samples were collected using a rosette multi-sampler mounted with probes and sensors for conductivity, temperature, and depth (Sea-Bird SBE 911Plus, Sea-Bird Electronics, United States). Subsamples (100 ml) were collected in HCL-rinsed bottles and stored at 4°C for nutrient analysis. Nutrient analysis was performed (in duplicate) on Technicon AA3 Auto-Analyzer (Bran+ Luebbe, Norderstedt, Germany) for phosphate (PO_4_^3−^), ammonium (NH_4_^+^), nitrite (NO_2_^−^), nitrate (NO_3_^−^), and silicic acid [Si(OH)_4_]. The detection limits of the Auto-Analyzer for each inorganic nutrient were; 0.024–39 μM (for phosphate), 0.04–27 μM (ammonium), 0.003–6 μM (nitrite), 0.015–50 μM (nitrate), and 0.03–100 μM (silicic acid). For analysis of Chlorophyll (Chl-*a*), 500 ml of seawater was vacuum filtered (<10 mm Hg) through Whatman GF/F filter membranes (25 mm), packed carefully in aluminum foil, and stored at −20°C in the dark until further analysis. Chl-*a* was extracted with the 90% acetone method and analyzed using a fluorometer (CHL NA, Model # 046, Turner designs, San Jose, CA, United States). For molecular analysis, 2 L seawater was filtered on a 0.22 μm GTTP filter (Millipore, Eschborn, Germany). The filters were flash-frozen in liquid nitrogen, transferred to the laboratory, and stored at −80°C until DNA extraction.

### DNA Extraction and Amplification of Form ID *rbc*L Gene

The genomic DNA was extracted using the DNeasy PowerWater DNA extraction kit (QIAGEN, Hilden, Germany) according to the manufacturer’s instruction. Further quality and quantity of DNA were checked on 1% agarose gel electrophoresis (Thermo Fisher Scientific, Wilmington, Delaware, United States). Form ID *rbc*L gene (554 bp), fragments were amplified using the previously published *rbc*L primer (Wawrik et al., 2002). Each PCR reaction was performed using the following reaction ingredients: 2 μl template DNA, 10 μl Premix Taq (Takara, Tokyo, Japan), 1 mM each primer, and 6 μl of double-distilled water to make a final volume of 20 μl. Further, while performing PCR, conditions were set as follows: initial denaturation at 95°C for 5 min, 30 cycles of 95°C for 1 min, 56°C for 1 min, 72°C for 1 min, and a final extension at 72°C for 1.2 min. PCR reactions of environmental samples were performed in triplicate, pooled together, and purified using Universal DNA purification kits (Tiangen Biotech, Beijing, China) following the manufacturer’s instruction. All libraries were constructed and sequenced *via* a paired-end approach (PE300) on an Illumina MiSeq PE300 platform (Illumina, San Diego, CA, United States) at Allwegene Technology Co. Ltd. Beijing, China.

### Data Processing and Statistical Analysis

Raw sequences were obtained from the Illumina Miseq PE300 platform, which then transformed into sequence reads by base calling using the Illumina Analysis Pipeline (v2.6). These sequences were stored in FASTQ files with respective sequencing quality. Based on samples and their barcodes, raw sequence data were separated, permitting up to one mismatch. Further, open-source software QIIME (v1.8; Caporaso et al., 2010) was used to quality filter the raw sequence. According to the relation between paired-end reads, the paired-end was merged into full-length sequences by FLASH software (v1.2.7), and a minimum overlap of 10 bp length was kept. The maximum mismatch ratio allowed by the overlap was kept at 0.1. Every sample raw tag was quality filtered to obtain clean tags by Trimmomatic software (v0.33; Bolger et al., 2014), and sequences meeting the following three criteria were included in downstream analyses: (1) sequences with precise primers and bar-codes; (2) quality score >30; (3) sequences >200 bp in length. The sequencing quality score is based on the probability that the base is called incorrectly. The Q30 quality score can be inferred with 1 in 1000 probability of an incorrect base call, which corresponds to 99.99% base call accuracy. A sequence length >200 was kept as *rbc*L gene length is 554 bp. The sequencing platform that we used was Illumina MiSeq PE300. Illumina reads are relatively shorter (150–300 bp) compared to the *rbc*L gene of 554 bp. This shortcoming was addressed by the paired-end sequencing that covered the 554 bp *rbc*L gene. Further, To eliminate erroneous and chimeric sequences, USEARCH (v10.0.240; Edgar, 2010) was used. After removing non-*rbc*L sequence reads, sequences were clustered into operational taxonomic units (OTUs) at a 97% similarity level using UCLUST (v1.2.22). Different methods use a defined yet arbitrary clustering threshold, called the sequence similarity threshold, as a cutoff value to ensure that the sequence within OUTs is identical. We used a 97% cutoff value, which can effectively maximize genetic diversity. Low-abundance OTUs (fewer than two reads, including singletons), which might influence richness and diversity estimates, were excluded from the subsequent analyses (Dickie, 2010). The remaining high-quality sequences were queried against the GenBank database at NCBI using local BLASTn. The MEGAN program (Huson et al., 2007) was used to assign BLAST hits to taxa in the NCBI database. A phylogenetic tree was constructed based on top genera recovered from this study. Prior to construction of the phylogenetic tree, top genus sequences were first translated to amino acid sequences. These amino acid sequences were then blasted in the protein database at National Center for Biotechnology Information (NCBI) using BLASTX (v2.8.1+) to identify the most closely related sequences (Altschul et al., 1997). These sequences were then further aligned with ClustalW, and a phylogenetic neighbor-joining tree was constructed using MEGA (v7.0; Huson et al., 2007; Kumar et al., 2016). Later, the cluster stability was verified by bootstrap resampling for 1,000 times. Further, this phylogenetic tree was edited with online webpage iTOL (Letunic and Bork, 2011). The sequences obtained from this study have been deposited in the NCBI Sequence Read Archive with accession number SUB6119769.

Chao1 (richness estimator) and Shannon diversity indices were calculated using QIIME (v1.8). The coverage of sequencing and abundance was calculated by a random sampling method (mothur), and the rarefaction curve was drawn using R (v3.3.1). In the present study, nonmetric multidimensional scaling (NMDS) was used to show vertical and horizontal distribution outline of the chromophytic community using Primer (v6; Clarke and Gorley, 2006). Before NMDS analysis, data were log-transformed in primer software, and clusters were overlaid using a resemblance matrix of Bray-Curtis similarity. To understand the spatial distribution of chromophytic phytoplankton and their relationship within environmental parameters, we performed redundancy analysis (RDA). Before RDA analysis, detrended correspondence analysis (DCA) was carried out to know whether RDA or canonical correspondence analysis is suitable for the current study. Since the length of the first axis was less than 2.0, RDA was selected. The differences of various environmental parameters with different depths were evaluated by *t*-test using Excel (Command TTEST, two-tailed).

## Results

### Environmental Variability

**Figure 2:**
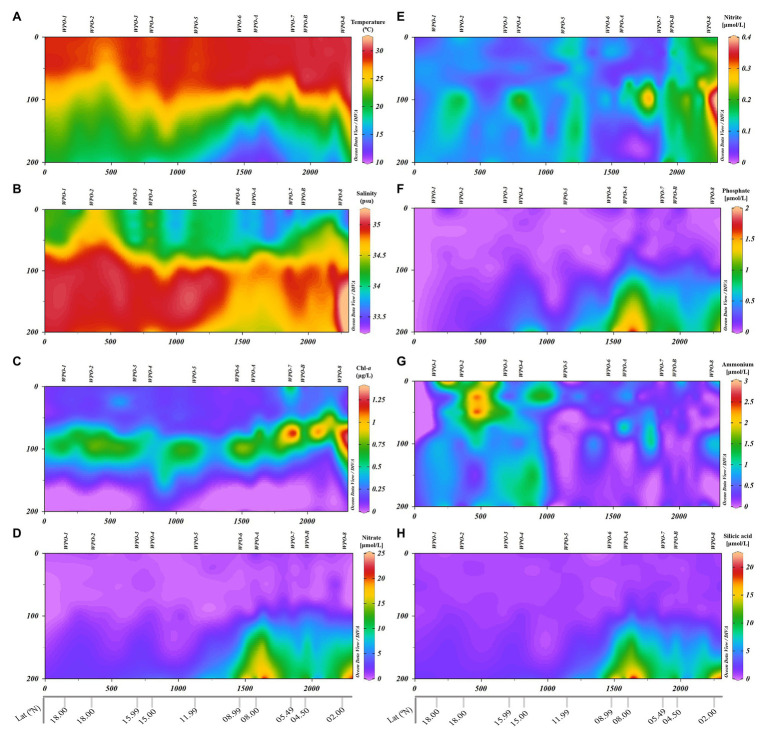
Vertical depth profiles of environmental parameters measured in the WPO. **(A)**: Temperature (°C), **(B)**: Salinity-*a*, **(C)**: Chl-*a*, **(D)**: Nitrate (NO_3_^−^), **(E)**: Nitrite (NO_2_^−^), **(F)**: Phosphate (PO_4_^3−^), **(G)**: Ammonia (NH_4_^+^), and **(H)**: Silicic acid [Si(OH)_4_].

The oceanographic setup had a strong bearing on the environmental characteristics in the WPO (Figure 2). Regional differentiation in the surface environmental variables was observed (two-tailed *t*-test) within the northern stations (WPO-1–WPO-5) and equatorward (southern) stations (WPO-6–WPO-8, WPO-A, and WPO-B). During the study period, Sea Surface Temperature (SST) and Sea Surface Salinity (SSS) values ranged from 27.9 to 29.9°C and 33.4 to 34.6, respectively. The lowest SST was recorded in the northern study area, especially at 15°N (station WPO-4; 27.9°C), whereas towards the equator, SST increased up to 29.9°C (at 4.5°N; station WPO-B). Contrastingly, high SSS was observed in the northern region, at 18°N (station WPO-2; 34.6), which eventually decreased (33.4) towards the south (5.4°N, at station WPO-7). Surface Chl-*a* concentration was higher (0.65 μg L^−1^) in the upwelling zone (station WPO-7), which substantially decreased (up to 0.14 μg L^−1^) towards the northern non-upwelling region. The surface dissolved nutrients, except phosphate showed an unequal distribution pattern (*p* > 0.05) along the study area. Surface phosphate concentration was below the detection limit at station WPO-1 (18°N), WPO-4 (15°N), WPO-5 (11.9°N), and WPO-8 (2°N), whereas the highest concentration was recorded at WPO-6 (8.9°N; 0.11 μmol L^−1^). The low surface nitrite concentration (0.05 μmol L^−1^) was observed at stations WPO-3 (15.9°N), WPO-4 (15°N; 0.06 μmol L^−1^) and WPO-7 (5.4°N; 0.05 μmol L^−1^), whereas highest values were recovered from station WPO-B (4.5°N; 0.19 μmol L^−1^), WPO-8 (2°N; 0.17 μmol L^−1^), and WPO-A (8°N; 0.10 μmol L^−1^). Similarly, surface nitrate concentration was high (2.01 μmol L^−1^) at southern area 8°N (station WPO-A) and WPO-B (4.5°N; 1.19 μmol L^−1^), and the lowest (0.01 μmol L^−1^) concentration reported at WPO-4 (15°N), WPO-5 (11.9°N; 0.46 μmol L^−1^), and WPO-6 (8.9°N; 0.46 μmol L^−1^). Surface ammonia concentration was below the detection limit at stations WPO-5 (11.9°N) and WPO-7 (2°N), whereas the highest concentration was recorded at WPO-2 (18°N; 1.09 μmol L^−1^). The highest silicic acid concentration was recorded at stations WPO-7 (5.49°N; 1.17 μmol L^−1^) and WPO-8 (2°N; 1.02 μmol L^−1^), whereas the lowest was recorded at WPO-6 (8.9°N; 0.46 μmol L^−1^).

The vertical profile of the temperature and salinity evinced the presence of two distinct water masses in the region (Figure 2). The equatorial surface water (ESW) was characterized by high temperature and low salinity, whereas equatorial subsurface water (ESSW) was characterized by lower temperature and higher salinity. Towards the northern stations (18°N–11.9°N), strong stratification was evidenced in the upper layers till ~75 m depth. The ESW above the thermocline was nutrient depleted as a consequence of stratification, whereas below the thermocline (in the ESSW), the nutrient concentration was moderate. This restricted the Depth Chlorophyll Maximum (DCM) in between the 75 m and 100 m depth in the non-upwelling stations.

The influence of upwelling signatures was clear on vertical profiles of physicochemical features in the southern upwelling zone (8.9°N–4.5°N) stations, i.e., WPO-6–WPO-8, WPO-A, and WPO-B (Figure 2). Eddy-driven upwelling transported cold, high-saline, and nutrient-enriched water towards the surface layer. This upward intrusion eventually decreased temperature and increased salinity upward from 200 m to ~100 m depth in the upwelling zone (*p* < 0.01; Figures 2A,B). Similarly, the dissolved inorganic nutrients (except nitrite and ammonia) concentration increased due to vertical transport of enriched water from 200 m to ~100 m depth (*p* < 0.01; Figures 2D,F,H). However, nitrite and ammonia concentration were unevenly dispersed in the upwelling zone (Figures 2E,G). The nutrient pumping from the deeper depths eventually supported the phytoplankton growth as revealed by Chl-*a* signatures. This significantly resulted (*p* < 0.05) in shallowing of DCM (~75 m) in the upwelling and neighboring stations (Figure 2C).

### Sequencing Statistics and Estimates of Diversity Indices

A total of 1,476,652 raw sequences were generated from different vertical depth samples. Among 1,476,652 raw sequences, 312,108 clean sequences were included in the downstream analysis, and the details are given in Tables 1 and 2. Table 1 represents the sequencing data of stations (WPO-1 to WPO-8), which were sampled for three depths (0 m, 75 m, and 150 m). Table 2 represents two stations (WPO-A and WPO-B), which were sampled for five depths (0 m, 50 m, 75 m, 100 m, and 150 m). As we recovered a large number of OTUs, here, only OTUs with an abundance of more than 10 were considered for further analysis. Therefore, based on 97% similarity, we included a total of 755 OTUs. Further, the Shannon-Weiner diversity (H´) index was calculated for all samples. The highest diversity (6.67) was observed at 2°N, station WPO-8 (75 m), whereas the lowest value (3.67) was recorded at 4.5°N, station WPO-B (100 m; Tables 1 and 2). Chao 1 (OTU richness) was observed highest at station WPO 8 (75 m), and the lowest Chao 1 value was recorded at WPO-A (0 m; Tables 1 and 2). The observed species and Goods coverage were also listed in Tables 1 and 2.

**Table 1:** Summary of OTU numbers and diversity indices for *rbc*L sequences recovered from the various depths of the WPO stations WPO-1 to WPO-8.

Sites		WPO-1	WPO-2	WPO-3	WPO-4	WPO-5	WPO-6	WPO-7	WPO-8
Surface (0 m)	No. of OTUs	399	477	508	499	486	497	484	-
	Raw tags	46136	57290	81868	74410	19980	79214	39952	-
	Final tags	10068	10068	10068	10068	10068	10068	10068	-
	Observed species	385.5	459.2	490.7	480.8	469.6	478.8	466.4	-
	Chao1	534.68	661.14	648.48	667.65	652.87	678.02	657.17	-
	Good’s coverage	0.98	0.98	0.98	0.98	0.98	0.98	0.98	-
	Shannon-weiner	5.08	5.28	5.36	5.71	5.46	5.3	5.64	-
DCM (75 m)	No. of OTUs	482	436	435	588	513	526	462	917
	Raw tags	52514	22804	12012	19424	41414	23453	52581	44905
	Final tags	10068	10068	10068	10068	10068	10068	10068	10068
	Observed species	465	416.3	419.6	568.1	495.2	507.3	441.2	884.4
	Chao1	646.13	681.31	590.84	739.74	715.73	676.18	661.54	1139.88
	Good’s coverage	0.98	0.97	0.98	0.98	0.97	0.98	0.98	0.96
	Shannon-weiner	5.65	4.39	4.58	6.66	5.39	5.48	4.53	6.67
Bottom (150 m)	No. of OTUs	520	585	656	476	515	514	-	-
	Raw tags	62191	29585	24453	84994	30019	17038	-	-
	Final tags	10068	10068	10068	10068	10068	10068	-	-
	Observed species	498.2	561.1	633	458	491.5	493.3	-	-
	Chao1	744.05	839.72	900.19	624.33	782.02	775.94	-	-
	Good’s coverage	0.97	0.97	0.97	0.98	0.97	0.97	-	-
	Shannon-weiner	4.8	5.2	5.78	4.72	4.17	4.93	-	-

Archive with accession number SUB4422106**.

**Table 2:** Summary of OTU numbers and diversity indices for *rbc*L sequences recovered from the various depths of the WPO stations WPO-A to WPO-B.

Sites		WPO-A	WPO-B
Surface (0 m)	No. of OTUs	391	543
	Raw tags	68709	29508
	Final tags	10068	10068
	Observed species	376.8	518.2
	Chao1	493.42	794.42
	Good’s coverage	0.98	0.97
	Shannon-weiner	4.79	5.48
50 m	No. of OTUs	546	507
	Raw tags	55416	69512
	Final tags	10068	10068
	Observed species	524.5	487.5
	Chao1	746.22	694.91
	Good’s coverage	0.97	0.98
	Shannon-weiner	5.57	5.12
DCM (75 m)	No. of OTUs	533	400
	Raw tags	26617	22350
	Final tags	10068	10068
	Observed species	512.4	384.2
	Chao1	748.9	561.48
	Good’s coverage	0.97	0.98
	Shannon-weiner	5.59	4.63
100 m	No. of OTUs	481	428
	Raw tags	61819	21032
	Final tags	10068	10068
	Observed species	464.3	407.6
	Chao1	641.4	666.76
	Good’s coverage	0.98	0.97
	Shannon-weiner	5.11	3.67
Bottom (150 m)	No. of OTUs	359	601
	Raw tags	88026	117426
	Final tags	10068	10068
	Observed species	342.8	578.1
	Chao1	509.67	831.83
	Good’s coverage	0.98	0.97
	Shannon-weiner	3.74	5.12

### Community Composition and Phylogenetic Analysis of Chromophytic Phytoplankton

**Figure 3:**
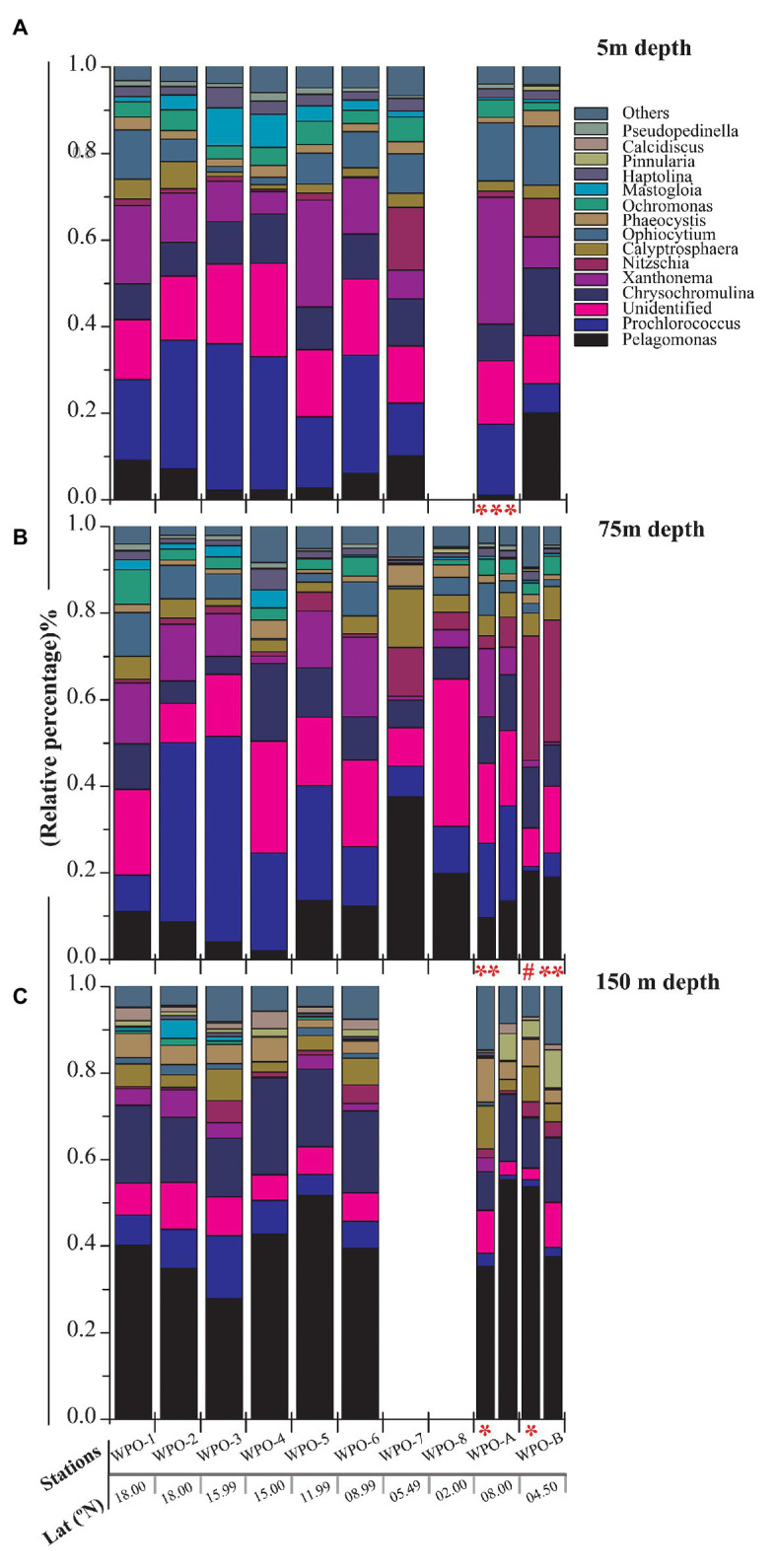
Community structure of chromophytic phytoplankton at genus level based on *rbc*L gene sequences. **(A)** represents 5m depth, **(B)** represents 75m depth and **(C)** represents 150m depth (Note: Blank column indicate no data. Other marks indicate extra depth sampling at respected stations: ^***^5 m; ^**^50 m; ^#^25 m; ^*^100 m).

**Figure 4:**
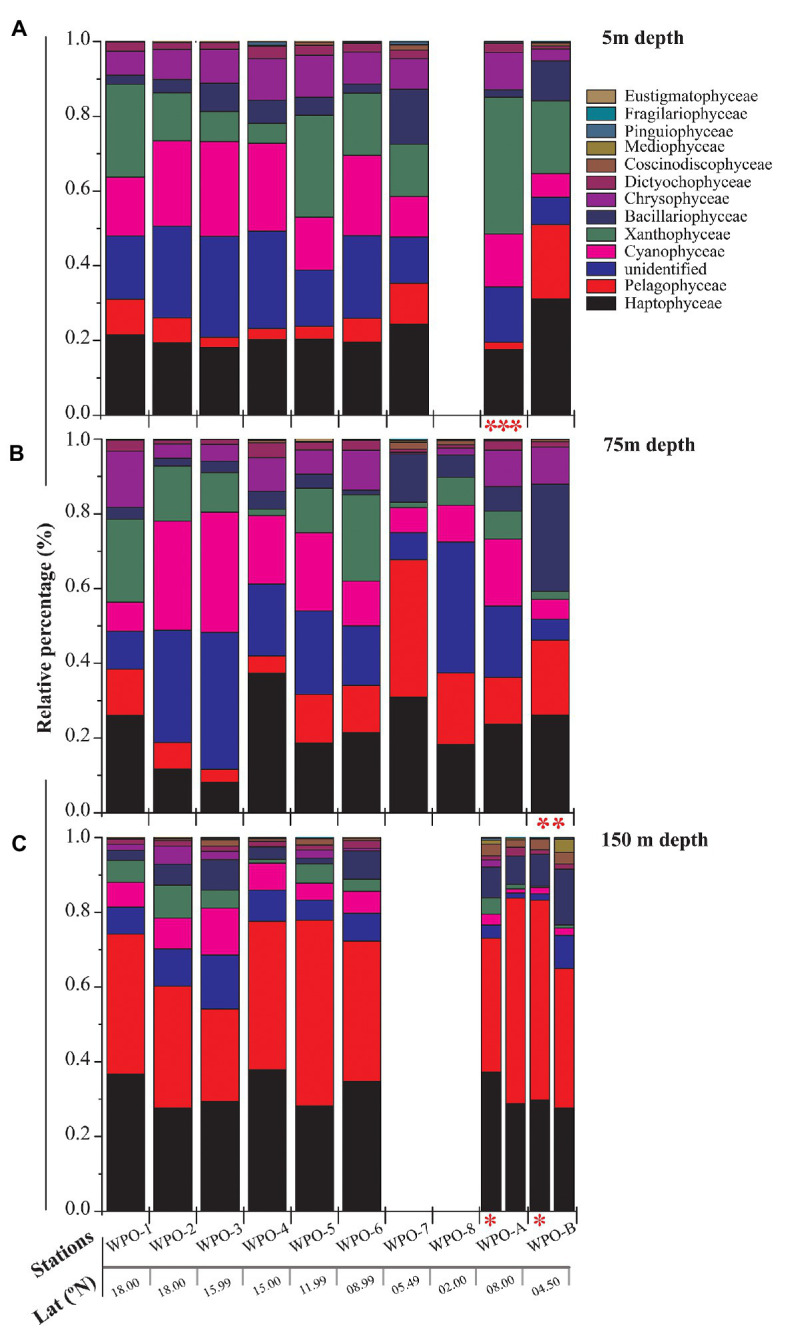
Community structure of chromophytic phytoplankton at class level based on *rbc*L gene sequences. **(A)** represents 5m depth, **(B)** represents 75m depth and **(C)** represents 150m depth (Note: Blank column indicate no data. Other marks indicate extra depth sampling at respected stations: ^***^5 m; ^**^50 m; ^*^100 m).

All major chromophytic phytoplankton groups containing form ID *rbc*L gene, such as Bacillariophyceae, Haptophyceae, Pelagophyceae, Pinguiophyceae, Xanthophyceae, Eustigamtophyceae, Cyanophyceae, Syrunophyceae, Chrysophyceae, and Dictyochophyceae, were detected. The most abundant genera and classes are shown in Figures 3, 4, respectively. In general, the relative abundance at the class level revealed the dominance of the Haptophyceae rather than the Bacillariophyceae (Figure 4). At the genera level, *Pelagomonas* and *Prochlorococcus* outnumbered other chromophytic phytoplankton (Figure 3).

**Figure 5:**
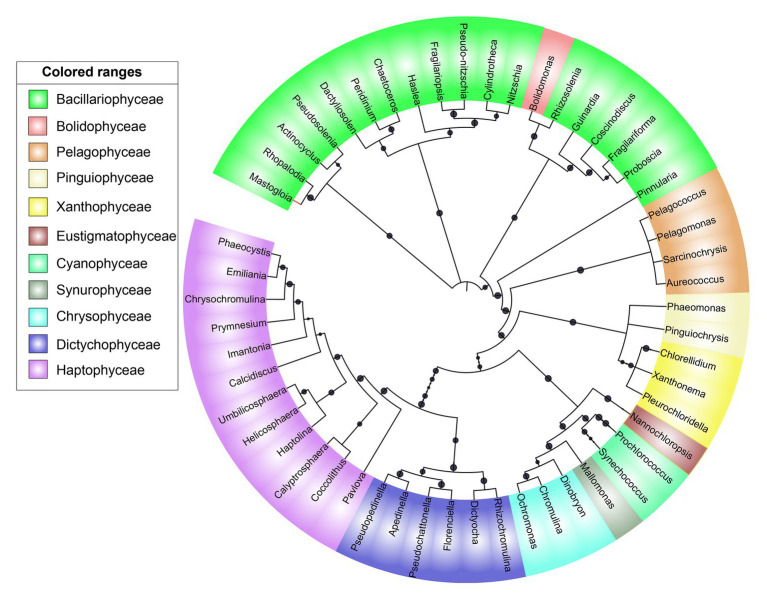
A neighbor-joining phylogenetic tree constructed based on *rbc*L amino acid sequences. The topology of the tree was inferred from 1,000 bootstrap resampling, and bootstrap values greater than 50% were labeled with black dots at branches.

A neighbor-joining phylogenetic tree was constructed based on the *rbc*L gene amino acid sequences (Figure 5). Bacillariophyceae was retrieved as the most diverse group (with a total of 18 OTUs), than the dominant Haptophyceae group. Among Bacillariophyceae, *Nitzschia* was the dominant genus recovered at 4.5°N (station WPO-B) 50 m depth (Figure 3B). Within Bacillariophyceae, genus *Bolidomonas* (Class-Bolidiophyceeae) was clustered with *Rhizosolenia*, which is a genus of class Bacillariophyceae (Figure 5). *Bolidomonas* are picoplanktonic flagellated algae, which have a symbiotic relationship with diatoms (Kuwata et al., 2018). Further, Haptophyceae recovered as a second most diverse group, represented by a total of 12 OTUs, among which *Chrysochromulina* was dominant at 15°N (station WPO-4) 150 m depth followed by *Calyptrosphaera* at 5.4°N (station WPO-7) 75 m depth (Figures 3B,C). Class Pelagophyceae was represented by four genera, including *Pelagococcus*, *Pelagomonas*, *Sarcinochrysis*, and *Aureococcus*. The dominance of *Pelagomonas* has been recorded at 8°N (station WPO-A) 150 m depth, whereas the lowest value was recorded at the same location in surface waters (Figures 3A,C). Class Cyanophyceae represented by *Prochlorococcus* and *Synechococcus*, wherein *Prochlorococcus* was recorded as the most dominant genus at 15.9°N (stations WPO-3) at 75 m (Figure 3B). Other classes, including Dictyochophyceae (6 OTUs), Xanthophyceae (3 OTUs), Piguiophyceae (2 OTUs), Bolidiophyceae (1 OTU), Synurophyceae (1 OTU), and Eustigmatophyceae (1 OTU) were also recovered in the study region (Figure 5).

### Effect of Environmental Variables on Chromophytic Phytoplankton

**Figure 6:**
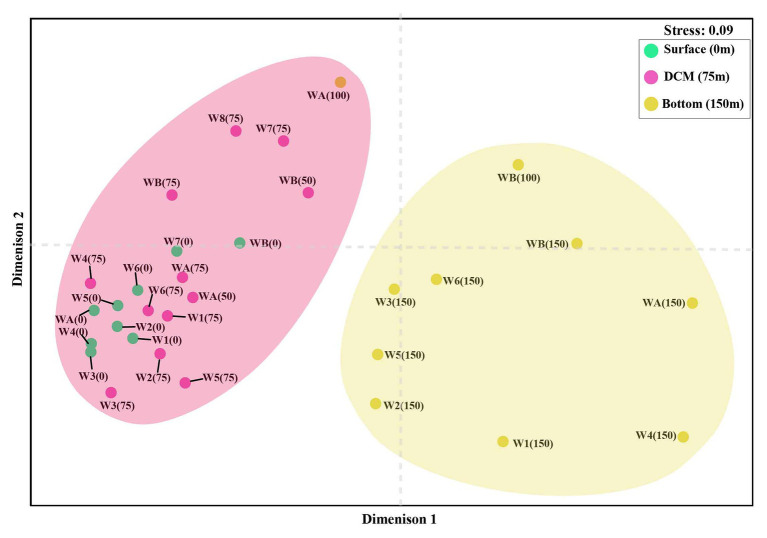
Non-metric multidimensional scaling (NMDS) analysis of chromophytic phytoplankton community (stress = 0.09, similarity = 50%) from the WPO. [W1: WPO-1(18°N), W2: WPO-2 (18°N), W3: WPO-3 (15°N), W4: WPO-4 (15°N), W5: WPO-5 (11°N), W6: WPO-6 (8.9°N), W7: WPO-7 (5.4°N), W8: WPO-8 (2°N), WA: WPO-A (8°N), WB: WPO-B (4.5°N)].

The nonmetric multidimensional scaling (NMDS) separated clusters of surfaces, DCM, and 150 m depths on the basis of 50% similarity (Figure 6). At the surface layers (0 m), the chromophytic phytoplankton community was relatively dominated by species belonging to Haptophyceae (Chrysochromulina, Calyptroshaera), Cyanophyceae (Prochlorococcus), and Xanthophyceae (Xanthonema, Ophiocytium) in both the non-upwelling and upwelling regions (Figures 3A, 4A). In the DCM depths (50 and 75 m), the relative percentage of Cyanophyceae (Prochlorococcus) was more than Haptophyceae (Chrysochromulina, Calyptroshaera) in the non-upwelling stations. However, towards the upwelling zones, the relative percentage of Cyanophyceae was suppressed by Haptophyceae (Chrysochromulina, Calyptroshaera) (Figures 3B, 4B). At the deeper depths, Pelagophyceae were relatively dominated over the Haptophyceae in non-upwelling (150 m) as well as upwelling zones (100 m and 150 m) (Figures 3C, 4C). Notably, Bacillariophyceae was observed in-between DCM and 150 m depth in the upwelling zone (Figures 4B,C). Contrary, Xanthophyceae, and Chrysophyceae were relatively more on surface than deeper waters in both the non-upwelling and upwelling zone (Figures 3 and 4).

**Figure 7:**
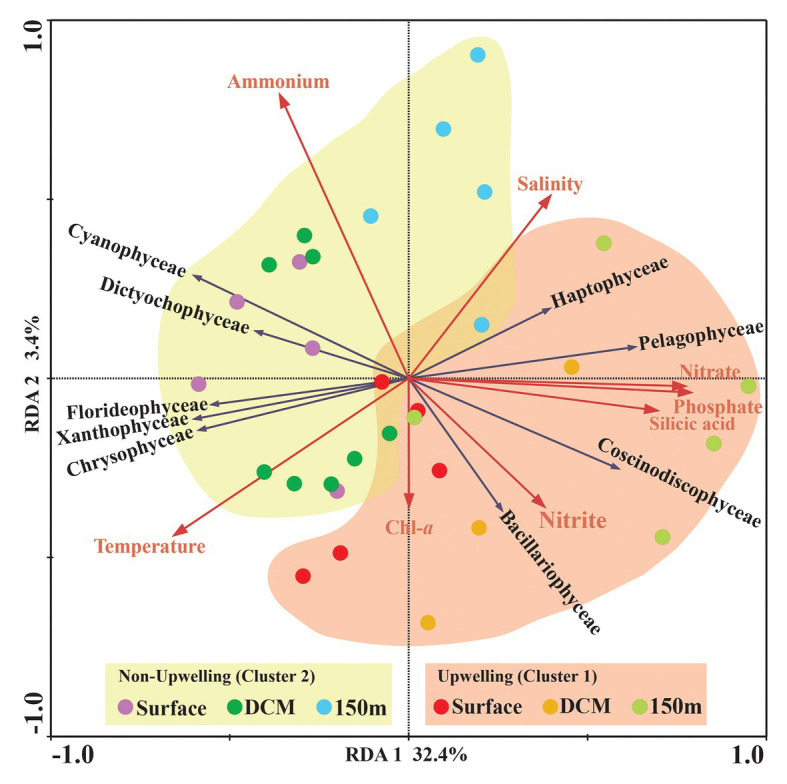
Redundancy analysis (RDA) for *rbc*L gene-based chromophytic phytoplankton community distribution and environmental factors, the black arrow represented different classes of phytoplankton whereas environmental variables were shown with red arrows. Correlation between environmental variables [Temperature (°C), Salinity, Chl-*a*, Nitrate (NO_3_^−^), Nitrite (NO_2_^−^), Phosphate (PO_4_^3−^), Ammonia (NH_4_^+^) and Silicic acid [Si(OH)_4_] and RDA] axes are shown by both length and angle of arrows. The two RDA axes explained 34.8% of the total variation.

**Figure 8:**
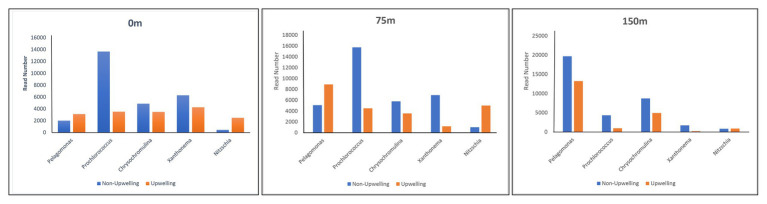
Rarefaction curves comparing the number of reads to the number of phylotypes (OTUs) found in the WPO samples.

The comprehensive relationship between the chromophytic phytoplankton and environmental variability was evaluated with Redundancy analysis (RDA; Figure 7). Orientation and length of environmental vectors indicated their relative importance and approximate relations to the chromophytic phytoplankton assemblage. In the RDA triplot, the first two axes explained >35.8% cumulative variation between chromophytic phytoplankton community and environmental variables. Further, the orientation of the sampling stations on an RDA triplot reflected their chromophytic phytoplankton assemblage and associated with the environmental variables. Station orientation defined two distinct clusters representing the upwelling (cluster 1) and non-upwelling (cluster 2) sampling depths. At deeper sampling depths (DCM and 150 m) in the upwelling zone (cluster 1) salinity and nutrients (nitrate, phosphate, and silicic acid) supported the relative abundance of the Pelagophyceae and Haptophyceae. Moreover, Bacillariophyceae is significantly influenced by the nitrite concentration together with the other nutrients (Figure 7). In cluster 2, surface and DCM depths from the non-upwelling zone were oriented opposite to salinity and nutrients, towards the temperature vector. The more relative percentage of Cyanophyceae, Xanthophyceae, and Chrysophyceae in these samples could be resultant of the low nutrient conditions. Furthermore, the orientation of surface samples from cluster 1 towards the temperature vector could be due to the dominance of low nutrient and warmer temperature preferring chromophytic phytoplankton communities, i.e., Cyanophyceae, Xanthophyceae, and Chrysophyceae. The 150 m depths of Cluster 2 were oriented towards the salinity vector due to the dominance of Pelagophyceae and Haptophyceae. The plateaued rarefaction graph is drawn and shown in Figure 8.

## Discussion

For the last two decades, *rbc*L genes have proven to be an important and reliable phylogenetic marker for deciphering the diversity of chromophytic phytoplankton. Previous studies based on a large subunit of *rbc*L gene have recognized its significance in decrypting the community structure of chromophytic phytoplankton from different geographical and ecological settings in the global oceans (summarized in Table 3). However, to date, the distribution and responses of the chromophytic phytoplankton community to the ecological niche in the WPO has not been evaluated. In the WPO, diverse WBC-induced eddies and upwelling zones provide a unique environment for the proliferation of the marine phytoplankton community. Earlier phytoplankton studies in this region were restricted to satellite-derived ocean color imagery, microscopy, and flow cytometer evaluation of species composition in response to physicochemical characteristics (Chen et al., 2017, 2018). The only study on the functional *nif* H gene evaluation was focused on a wide distribution of diazotrophic community and quantification in the WPO (Chen et al., 2019). Here, we present the first study based on functional gene *rbc*L of the chromophytic phytoplankton with relation to the regional environmental characteristics (eddies and upwelling) in the WPO.

**Table 3:** Comparison of chromophytic phytoplankton community reveled using *rbc*L gene.

Type of gene	Location	Date	Dominant genus/Class	Reference
*rbc*L Type I	Eastern Gulf of Mexico	August 1994 and Sept 1993	*Prochlorococcus* sp., *Synechococcus* sp.	Pichard et al., 1993
*rbc*L Form ID	Monterey Bay and Western English Channel	August 1998 and July 1999	Pelagophyceae, Bacillariophycea and Raphidophyceae	Bhadury and Ward, 2009
*rbc*L Form ID	North Pacific Subtropical Gyre	2007–2009	Diatoms, prymnesiophytes and pelagophytes	Li et al., 2013
*rbc*L Form ID	Northern Gulf of Mexico	July 2005	Diatoms and other heterokonts	John et al., 2007
*rbc*L Form ID	Bering Sea	September 2009	Bacillariophycea and other eukaryotes	Endo et al., 2015
*rbc*L Form ID	Sundarbans Mangroves reserve forest	2010–2011	Bacillariophyceae, Cryptophyceae, Haptophyceae, Pelagophyceae, Eustigmatophyceae, and Rhaphidophyceae	Samanta and Bhadury, 2014
*rbc*L Form ID	Northern South China Sea	August 2007	Bacillariophyceae, Haptophyceae and Cyanophyceae	Li et al., 2016
*rbc*L Form ID	Bay of Bengal	Nov-Dec 2016	Pelagophyceae, Cyanophyceae, and Haptophyceae	Pujari et al., 2019
*rbc*L Form ID	West Pacific Ocean	November 2017	Haptophyceae, Pelagophyceae, Cyanophyceae, Bacillariophyceae and Chrysophyceae	Present Study
*nif* H	East Indian Ocean	April 2017	Cyanobacteria (*Trichodesmium* spp.) Proteobacteria (Alpha, Beta, and Gamma)	Wu et al., 2019
*nif* H	South China Sea, Western Equatorial Pacific Ocean, Philippine Sea	July 2015	Cyanobacteria (*Trichodesmium*), Unicellular cyanobacterium (UCYN-B)	Chen et al., 2019
PBS gene, pcb gene, isiA gene	North American East Coast, Caribbean Sea, Eastern Tropical Pacific, Tropical South Pacific	--	*Synechococcus*, *Prochlorococcus*	Bibby et al., 2009

The phylogenetic analysis in this study recovered *Bolidomonas*-like *rbc*L sequences belonging to class Bolidiophyceae, which were clustered with *Rhizosolenia* (Class‐ Bacillariophyceae). In an earlier study, isolated strains of *Bolidomonas* using nuclear, plastidial, and mitochondrial gene markers compared *Bolidomonas* and *Triparma*, which later also included *Parmales* (Bolidiophyceae; Ichinomiya et al., 2016). Furthermore, the phylogenetic assessment revealed that *Parmales* were closely related to diatoms (Bacillariophyceae) and ubiquitously distributed but constituted a minor component of the phytoplankton community (Ichinomiya et al., 2016). Similarly, another genus, *Peridinium*-like *rbc*L sequences, which represent the class Dinophyceae, was clustered in the phylogenetic tree with *Chaetoceros* (Class‐ Bacillariophyceae; Figure 5). The SSU rDNA investigation in two *Peridinium* species (*P. balticum* and *P. foliaceum*) suggested that the ancestors of these dinoflagellates engulfed pennate diatoms during tertiary endosymbiosis event (Inagaki et al., 2000), and genus *Peridinium* thus showed a close affinity to diatoms *rbc*L gene sequence in the present study.

During our study, the high-throughput sequencing analysis recovered 11 chromophytic phytoplankton groups (Figure 5) in 31 samples analyzed from the selected depths of 10 stations in the WPO. The recovery of these groups was consistent with previous studies carried out in different ecosystems of the world (Samanta and Bhadury, 2016). However, in contrast to previous studies, a dominance of group Haptophyceae over that of Bacillariophyceae was observed in this study. A total of 12 OTUs of group Haptophyceae were recovered during the analysis. Among them, the genus *Chrysochromulina* outnumbered other Haptophyceae genera recovered (Figure 3C). Genus *Chrysochromulina* sequences were recovered from most of the stations with varying depths of WPO. However, the highest number of *Chrysochromulina* sequences were retrieved from 150 m depth at all stations (Figure 3C). Haptophytes are one of the most diverse groups of picophototrophs in modern open oceans, and studies based on the analysis of SSU rDNA showed that the haptophytes were dominant in the pelagic and coastal ocean environments (Fuller et al., 2006; McDonald et al., 2007). A study carried out by McDonald (2007) concluded that, in the Gulf of Naples, >45% total and >70% eukaryotic chloroplast sequences were haptophytes in origin. Moreover, genus *Chrysochromulina* is believed to be ubiquitous in the marine environment and occupies up to 65% total number of nano-phytoplankton cells (Thomsen, 1994; Hajdu et al., 1996). The phylogenetic position of the majority of the picohaptophytes suggests that they are mixotrophic in nature, i.e., they are able to survive through the phototrophic regime with uptake and assimilation of organic nutrients (Nygaard and Tobiesen, 1993; Liu et al., 2009). Generally, it is believed that in nutrient and light-limited conditions, mixotrophy provides a competitive advantage (Pålsson and Granéli, 2004). A culture study carried out under controlled conditions suggested that *Chrysochromulina* species can feed on diverse small green flagellates. Their ingestion rate was inversely proportional to light intensity, and it changes in response to variation in light intensity and phosphate status (Jones et al., 1993). However, the occurrence of *Chrysochromuilna* species blooming with a larger chloroplast beneath the ice (off the coast of Finland) in low-light conditions suggests the complexity of mixotrophic nature within the genus *Chrysochromuilna* (Pintner, 1968; Niemi and Hallfors, 1974). Interestingly earlier studies proved that the vertical distribution of other haptophytes varies with change in temperature and light availability (Malinverno et al., 2003). Recently, Gran-Stadniczeñko (2017) observed the increased haptophyte diversity and abundance in the deep chlorophyll maximum (DCM) in Oslofjorden, Skagerrak, through 18 s rRNA and 28 s RNA evaluation. Therefore, different species of haptophytes probably respond differently to the vertically changing environmental characteristics in various regions. In the present study, the Haptophyceae oriented towards the dissolved nutrients vectors at the deeper depth upwelling stations (DCM and 150 m) in our RDA triplot (Figure 7). The highest abundance of Haptophyceae sequences from the depth of DCM and 150 m where nutrients were fairly high in concentration compared with the surface layer. Thus, here it can be concluded that under the light-limited conditions at deeper depths, along with the mixotrophy behavior, the availability of nutrients, high salinity, and low temperature conditions supported the growth of Haptophyceae, especially genus *Chrysochromulina*, as evinced in other studies. Few rarely occurring genera were also observed in the WPO during the present study. One such rare genera *Calyptrosphaera* belonging to the class Haptophyceae was reported in the deeper depths (75 m and 150 m; Figures 3B,C). The studies related to genus *Calyptrosphaera* mainly consisted of morphological examination (Klaveness, 1973; Nöel et al., 2004), and there is very little information available on the distribution or ecology of this genus. Thus, the distribution of such rare occurring genus *Calyptrosphaera* is difficult to assess. Nöel (2004) proposed that heterococcolith-bearing cells of genus *Calyptrosphaera* had strong adhesive ability, which supports their survival under a wide range of irradiance, temperature, and nutrient concentrations. It is possibly the changing life phase under the varying stress levels that facilitate their survival. Nonetheless, our study contributes to the general distribution and ecology of genus *Calyptrosphaera*. Similarly, the sequences of other genus *Pseudopedinella* belonging to Class‐ Dictychophyceae were recovered in moderate numbers at surface depths during this study. Studies based on distribution of genus *Pseudopedinella* in marine niches are very scarce. The genus *Pseudopedinella* was earlier considered a member of the class Chrysophyceae, whereas it has been included within the class Dcityochophyceae since the 1980s (Hibberd, 1986).

In the present study, the genus *Ochromonas* (strain-CCMP1393) belonging to the class Chrysophyceae was recovered from most of the surface (and at a depth of 75 m) of the WPO (Figures 3A,B). The experimental studies of Lie et al. (2018) suggested that *Ochromonas* (strain-CCMP1393) is phagotrophic phytoflagellate with different nutritional strategies (phagotrophic, mixotrophic, or phototrophic nutrition). The highest growth of *Ochromonas* (strain CCMP 1393) in the presence of light was due to the upregulation of genes such as those involved in photosynthesis, light harvesting, chlorophyll synthesis, and carbon fixation (Lie et al., 2018). Thus, here it can be hypothesized that the *rbc*L gene analysis can (which is directly related to carbon assimilation) provide sufficient correlation of *Ochromonas* (strain CCMP 1393) presence mostly in the euphotic layers. Nonetheless, this is the first report of *Ochromonas* (strain CCMP 1393) from the WPO waters, and it can probably serve as the basic dataset for upcoming studies.

**Figure 9:**
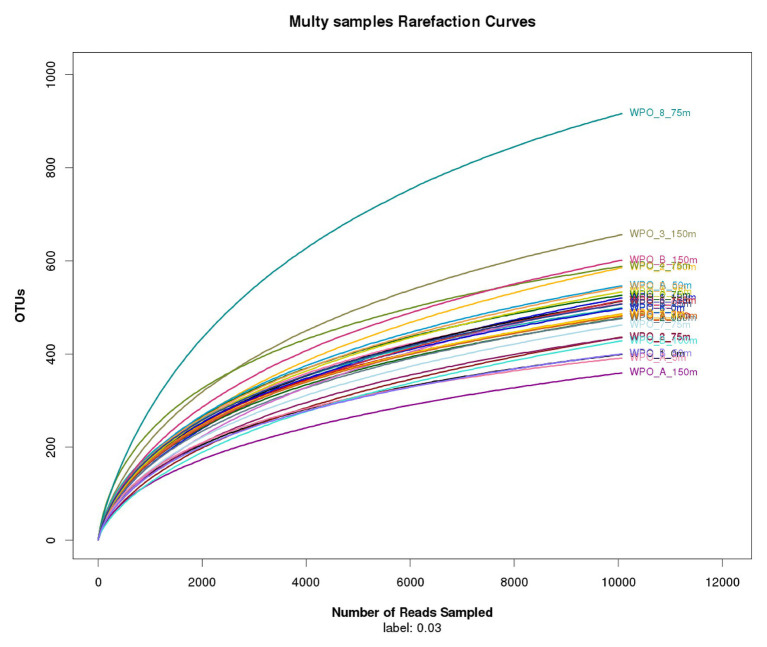
Distribution of dominant chromophytic phytoplankton species in Non-upwelling [WPO-1 (18°N), WPO-2 (18°N), WPO-3 (15.9°N), WPO-4 (15°N) and WPO-5 (11.9°N)], and upwelling [WPO-6 (8.9°N), WPO-7 (5.4°N), WPO-8 (2°N), WPO-A (8°N) and WPO-B (4.5°N)] region of the WPO.

During this study, a high abundance of class Pelagophyceae sequences, especially genus *Pelagomonas*, was recorded from both DCM and 150 m depths in the upwelling region, whereas it was observed more in number only at 150 m depth in non-upwelling regions of the WPO (Figure 9). Pelagophytes were recorded as major contributors to phytoplankton biomass and productivity in the various marine niches, including temperate, subtropical, and tropical open oceans (Suzuki et al., 2002; Ditullio et al., 2003; Cuvelier et al., 2010). Furthermore, they contribute to 10–20% of the total Chl-*a* in the equatorial Pacific Ocean. In the Northern Atlantic region, pelagophytes contributed a large fraction of phytoplankton biomass especially in the lower euphotic zone (Claustre and Marty, 1995). Previous evaluation of the form ID of the rbcL gene in coastal upwelling region of Monterey Bay by Bhadury and Ward (2009) also reported pelgophyte (such as *Pelagomonas calceolate*)-like sequences along with cosmopolitan haptophytes, including *Phaeocytis*, *chrysochromulina*, and *Emilania huxleyi*. The occurrence of this group was observed in coastal as well as open ocean upwelling regions of the WPO. Further, the cosmopolitan occurrence of these groups was also observed in the Sundarbans Mangrove Ecosystem (Samanta and Bhadury, 2014). Our previous study from the Bay of Bengal on the diversity of chromophytic phytoplankton using form ID rbcL gene showed the dominance of these groups in the southern area where wind-driven upwelling was likely the source of nutrients (Pujari et al., 2019). The orientation of Pelagophyceae in the RDA triplot at most of the deeper stations, especially in the upwelling zone, was highly correlated with most of the nutrients recorded during the study (Figure 7). This reveals the capability of Pelagophyceae species to utilize the nutrients at the deeper depths under light-limited conditions. The study carried out by Li et al. (2013) concluded with an *rbc*L gene study that these taxa can physiologically adapt to low light, nutrient-enriched conditions in the lower euphotic regions. However, in the upwelling regions of the WPO, the advection of nutrient-enriched deeper waters could better replenish and/or transport Pelagophyceae (especially *Pelagomonas*) population in the upper waters compared to the non-upwelling region.

A highly significant phytoplankton group for global marine biogeochemistry, Cyanophyceae, especially *Prochlorococcus*, was recorded in high abundance from most of the surface and DCM waters (Figure 9). However, recovery of Cyanophyceae-like sequences can be attributed to bias introduced by the primers used in this study for PCR amplification. During the evolution process, the *rbc*L gene gave rise to different forms. Form IA, IB, IC, and ID encode both green‐ and red-like RuBisCOs and present in different groups of phytoplankton (Watson and Tabita, 1997). Most dominant form IDs represent abundant groups such as Bacillariophyceae, Pelagophyceae, and Haptophyceae. Although, form IB encodes green lineages, including cyanobacteria (Paul et al., 1999). They mostly thrive in euphotic zones of the tropical and subtropical oligotrophic oceans, including the WPO (Chen et al., 2017) and adjacent Philippines sea (Gajigan et al., 2018). Here, Cyanophyceae (mainly *Prochlorococcus*) was evinced to occur in the euphotic zones of the surface and a depth of 75 m. These observations were also corroborated with earlier studies on RuBisCO large subunit gene probes to examine gene expression from the offshore waters of the Gulf of Mexico (Pichard et al., 1993), where the dominance of cyano, especially *Prochlorococcus rbc*L mRNA, reported at depths above 65 m. In the WPO, the occurrence of Cyanophyceae (mainly *Prochlorococcus*) in the euphotic zones were significantly correlated with the temperature and negatively correlated with most of the nutrients in RDA (Figure 7). Previous studies revealed that the *Prochlorococcus* population in phosphorus-limited environments contains more genes for phosphorus acquisition than the population where phosphorus is not a limiting factor (Martiny et al., 2006). Moreover, in oligotrophic oceans, the nitrogen-limited surface waters can be dominated by the High Light (HL) adapted strains, which have lower GC content and, therefore, may require less nitrogen to thrive. An amino acid encoded by low GC codons will have lower nitrogen (reduced N/C ratio) than those encoded by GC-rich codons. Therefore, we predict that the dominance of *Prochlorococcus* in surface and subsurface waters is probably attributed to the presence of HL strains, which are adapted to the high intensity of light.

## Summary

This study presents the first detailed investigation of a chromophytic phytoplankton community using high-throughput sequencing of *rbc*L genes in the WPO region. The variation observed in chromophytic phytoplankton suggests the strong influence of environmental variables induced by oceanographic features (eddy-induced upwelling) on the biological production in the WPO. The main chromophytic phytoplankton community signals recorded in the WPO are as follows; (1) a warmer, low-saline, and nutrient-imitated condition regulated the Cyanophyceae (mainly *Prochlorococcus* species) dominance at the surface and subsurface depths; (2) overall dominance of Haptophyceae, especially genus *Chrysochromulina*, under the light-limited conditions at deeper depths (DCM and 150 m) was probably influenced by the high salinity and fairly high dissolved nutrients. The mixotrophic mode of nutrition could also support the distribution of *Chrysochromulina* at deeper depths; (3) the capability of utilizing the nutrients under light-limited conditions supports the predominance of Pelagophyceae, especially the *Pelagomonas* species, in the deeper waters, and the advection of nutrient-enriched upwelled deeper waters could better replenish and transport the *Pelagomonas* population in the upper layers compared to the non-upwelling region of the WPO; (4) finally, compared to earlier studies, our comprehensive high-throughput sequencing analysis revealed some of the new and rare lineages, such as *Bolidomonas*, *Peridinium*, *Calyptrosphaera*, *Pseudopedinella*, and *Ochromonas* (strain-CCMP1393). Nevertheless, this is the first study to report these rare occurring genera in the WPO.

## Data Availability Statement

The datasets generated for this study can be found in NCBI SRA, https://www.ncbi.nlm.nih.gov/bioproject/PRJNA558162/.

## Author Contributions

LP designed the research, wrote the manuscript, and carried out the molecular and statistical analysis. DN and JK proofread and drafted the manuscript. CW helped in the experimental analysis. GZ, CD, and LL helped in sampling and proofread the manuscript. JS designed the research and drafted the manuscript. All authors contributed to the article and approved the submitted version.

### Conflict of Interest

The authors declare that the research was conducted in the absence of any commercial or financial relationships that could be construed as a potential conflict of interest.

## Footnotes

^1^https://odv.awi.de/
